# Proportion of preschool-aged children meeting the *Canadian 24-Hour Movement Guidelines* and associations with adiposity: results from the Canadian Health Measures Survey

**DOI:** 10.1186/s12889-017-4854-y

**Published:** 2017-11-20

**Authors:** Jean-Philippe Chaput, Rachel C. Colley, Salomé Aubert, Valerie Carson, Ian Janssen, Karen C. Roberts, Mark S. Tremblay

**Affiliations:** 1 Healthy Active Living and Obesity Research Group, Children’s Hospital of Eastern Ontario Research Institute, 401 Smyth Road, Ottawa, ON K1H 8L1 Canada; 2 Health Analysis Division, Statistics Canada, Ottawa, ON K1A 0T6 Canada; 3 Faculty of Physical Education and Recreation, University of Alberta, Edmonton, AB T6G 2H9 Canada; 4 School of Kinesiology and Health Studies, Department of Public Health Sciences, Queen’s University, Kingston, ON K7L 3N6 Canada; 5 Centre for Surveillance and Applied Research, Public Health Agency of Canada, Ottawa, ON K1A 0K9 Canada

**Keywords:** Physical activity, Sedentary behaviour, Screen time, Sleep, Obesity, Weight, Surveillance, Recommendations, Early years

## Abstract

**Background:**

New *Canadian 24-Hour Movement Guidelines for the Early Years* have been released in 2017. According to the guidelines, within a 24-h period, preschoolers should accumulate at least 180 min of physical activity (of which at least 60 min is moderate-to-vigorous physical activity), engage in no more than 1 h of screen time, and obtain between 10 and 13 h of sleep. This study examined the proportions of preschool-aged (3 to 4 years) Canadian children who met these new guidelines and different recommendations within the guidelines, and the associations with adiposity indicators.

**Methods:**

Participants were 803 children (mean age: 3.5 years) from cycles 2–4 of the Canadian Health Measures Survey (CHMS), a nationally representative cross-sectional sample of Canadians. Physical activity was accelerometer-derived, and screen time and sleep duration were parent-reported. Participants were classified as meeting the overall *24-Hour Movement Guidelines* if they met all three specific time recommendations for physical activity, screen time, and sleep. The adiposity indicators in this study were body mass index (BMI) z-scores and BMI status (World Health Organization Growth Standards).

**Results:**

A total of 12.7% of preschool-aged children met the overall *24-Hour Movement Guidelines*, and 3.3% met none of the three recommendations. A high proportion of children met the sleep duration (83.9%) and physical activity (61.8%) recommendations, while 24.4% met the screen time recommendation. No associations were found between meeting individual or combined recommendations and adiposity.

**Conclusions:**

Very few preschool-aged children in Canada (~13%) met all three recommendations contained within the *24-Hour Movement Guidelines*. None of the combinations of recommendations were associated with adiposity in this sample. Future work should focus on identifying innovative ways to reduce screen time in this population, and should examine the associations of guideline adherence with health indicators other than adiposity.

## Background

It is well established that a lifestyle characterized by high levels of physical activity, low levels of sedentary behaviour, and sufficient amounts of sleep is important for optimal health. Although movement-related behaviours (i.e., physical activity, sedentary behaviour, and sleep) have typically been studied in isolation, compelling evidence shows that these behaviours interact with one another to impact health [1–3]. In order to move from a segregated to an integrated approach to healthy movement behaviours for children in the preschool years (aged 0–4 years), a group of international experts undertook an evidence-informed process to develop and subsequently release in 2017 the *Canadian 24-Hour Movement Guidelines for the Early Years (0–4 years): An Integration of Physical Activity, Sedentary Behaviour, and Sleep* (*24-Hour Movement Guidelines*) [4]. The guidelines provide a new approach to population health promotion by integrating all behaviours along the movement continuum in order to shift the thinking from “independent movement behaviours” to the concept that “the whole day matters” [4].

Despite mixed findings in the literature, even isolated adherence to physical activity, screen time, and sleep duration recommendations has generally been associated with lower adiposity in children of the early years [5–7]. However, how combinations of these movement behaviours are associated with adiposity in this population is largely unknown. Given that 42 million children under 5 years have overweight or obesity worldwide [8] and that early intervention of movement behaviours has been identified as a promising strategy for obesity prevention [9], it is important to examine these associations from an integrated perspective.

The objectives of this study were twofold. First, we aimed to determine the proportion of preschool-aged children in Canada who meet the overall *24-Hour Movement Guidelines* and the individual, time-specific recommendations for physical activity, screen time, and sleep duration that are contained within the guidelines. Second, we aimed to examine the associations between meeting versus not meeting the physical activity, screen time, and sleep duration recommendations (and combinations of these recommendations), and body weight status and body mass index (BMI) z-scores.

## Methods

### Participants

Participants were children aged 3 to 4 years from cycles 2 (2009–2011), 3 (2012–2013) and 4 (2014–2015) of the Canadian Health Measures Survey (CHMS) [10]. Each cycle of the CHMS collects cross-sectional data from a nationally representative sample of the Canadian population aged 3–79 years living in private households. Data from cycle 1 were not included in the present paper because only 6- to 79-year-olds were surveyed in that cycle. For this analysis, data from cycles 2 to 4 of the CHMS were combined (according to Statistics Canada specifications) [11], in order to increase sample size and the precision of estimates.

Data collection included an interview in each participant’s home (conducted by trained Statistics Canada survey staff), followed by a visit by the participant to a mobile examination centre for a physical health examination. At the end of this examination, participants received an accelerometer to wear during waking hours for 7 consecutive days. A total of 1155 participants aged 3 to 4 years were eligible for this study. This analysis is based on a subset of that sample (*n* = 803); the remainder were excluded because of incomplete accelerometer (*n* = 330), measured BMI (n = 8), or household education (*n* = 14) data.

Ethics approval for the CHMS was obtained from Health Canada and the Public Health Agency of Canada Research Ethics Board [12]. Written informed consent was obtained from a parent or legal guardian, and assent was obtained from the child. More information about the CHMS can be found elsewhere [13–16].

### Meeting the *24-Hour Movement Guidelines*

For surveillance of the new *24-Hour Movement Guidelines* for preschoolers (aged 3–4 years), it has been agreed [4] that the following recommendations be examined to assess adherence: within a 24-h period, preschoolers should have at least 180 min of physical activity (of which at least 60 min is moderate-to-vigorous physical activity [MVPA]), engage in no more than 1 h of screen time, and obtain between 10 and 13 h of sleep. We classified participants as meeting the overall *24-Hour Movement Guidelines* if they met all of these recommendations.

#### Physical activity

Physical activity was objectively measured using Actical accelerometers (Philips Respironics, Oregon, USA). Participants wore the device on an elastic belt around their waist over their right hip for 7 consecutive days. Data were collected in 60-s epochs in cycle 2, and 15-s epochs in cycles 3 and 4. The memory capacity of the Actical accelerometers used in the CHMS allowed for 7 days of data to be recorded in cycle 2 (60-s epochs), but for only ~5.5 days for cycles 3 and 4 because a shorter epoch was used (15-s epochs); therefore, days 6 and 7 were excluded for participants in cycle 2 in order for data to be comparable across cycles. Data were collected on a continuous basis, 7 days per week, so excluding days 6 and 7 introduced no bias. Non-wear time in cycle 2 was defined as ≥60 consecutive minutes of zero counts, with allowance for 2 min of counts between zero and 100. Non-wear time in cycles 3 and 4 was defined as at least 240 intervals of 15 s of zero counts, with allowance for 30 s of counts between 0 and 25 [17, 18]. Daytime naps should thus be counted as non-wear time rather than sedentary time. A valid day was defined as ≥5 h of wear time during waking hours [18]. To be included in the analyses, participants were required to have 3 or more valid days [19, 20].

The mean wear time for valid days was 12.0 h in the combined cycles. In cycle 2, light-intensity physical activity (LPA) was defined as 100–1149 counts per minute (cpm) and MVPA as ≥1150 cpm [21]. In cycles 3 and 4, LPA was defined as 25–278 counts per 15 s and MVPA as ≥288 counts per 15 s. The 15-s epoch data of cycles 3–4 were then converted into min/day by multiplying the values by 4. In order to ensure the data were comparable across cycles, correction factors were used on the min/day variables from cycle 2 [22]. Average minutes per day of physical activity (i.e., ≥LPA) and MVPA across valid days were calculated, and participants were categorized as meeting the physical activity recommendation if they obtained ≥180 min/day of physical activity at any intensity, including at least 60 min of MVPA.

#### Screen time

Average daily screen time of participants was assessed as part of the in-home interview. Parents/guardians were asked, on average, how many hours a day the child spends 1) watching TV or videos or playing video games, and 2) on a computer. The response categories differed between cycle 2 (none, <1, 1 to 2, 3 to 4, 5 to 6, ≥7) and cycles 3–4 (none, <1, 1 to <3, 3 to <5, 5 to <7, ≥7). For cycle 2, the average daily screen time was derived using the mid-point of the response category (i.e., 0, 0.5, 1.5, 3.5, 5.5 and 7 h for the respective categories). For cycles 3 and 4, the same midpoints applied in cycle 2 were assigned to each of the respective categories for consistency, as previously shown [23]. Results of the two screen time questions were summed to determine average daily screen time. Participants were categorized as meeting the screen time recommendation if that sum was ≤1 h/day.

#### Sleep duration

Since accelerometers were worn only during waking hours, no objective measure of sleep was available in the CHMS. Average daily sleep duration of participants was therefore assessed by proxy report as part of the in-home interview. Parents/guardians were asked: “How many hours does your child usually spend sleeping in a 24-hour period, excluding time spent resting?” Responses were rounded to the closest half-hour by the interviewer. Participants were categorized as meeting the sleep duration recommendation if parents reported that their child obtained between 10 and 13 h of sleep a day.

### Adiposity

The adiposity indicators in this study were BMI z-scores and body weight status as defined by the World Health Organization Child Growth Standards [24]. Adiposity was measured in the same way for all three CHMS cycles. Body weight and height were directly measured according to standardized procedures by trained health measures specialists [25]. BMI (kg/m^2^) was calculated, and age- and sex-specific BMI z-scores were computed [24]. Participants were classified as “at risk of overweight, or overweight/obesity” or “not” for the analyses. Of note, there were no underweight (also referred as thin) children in this sample. It was not possible to create an overweight/obesity status-only group due to a lack of statistical power (only 8% of the sample had overweight or obesity).

### Covariates

Age, sex, and highest household education were included as covariates based on previous research examining the association between movement behaviours and health indicators in children of the early years [5–7] and availability of measures within the CHMS. Highest household education was coded into three categories: “secondary school or less”, “some post-secondary, less than bachelor’s degree”, and “bachelor’s degree or higher”.

### Statistical analyses

All analyses were performed using SAS version 9.3 (SAS Institute, Cary, NC, USA) and SUDAAN version 11.0 (RTI International, Raleigh, NC, USA). Survey and accelerometer-specific survey weights for the combined cycles were used to ensure results were representative of the Canadian population in this age group [11]. Descriptive statistics were used to examine the average daily time engaged in physical activity, screen time, and sleep as well as the proportion of participants meeting the *24-Hour Movement Guideline* recommendations for each movement behaviour both separately and for all possible combinations. Associations between meeting versus not meeting the recommendations of the *24-Hour Movement Guidelines*, alone or in combinations, with “at risk of overweight, or overweight/obesity” (0, no; 1, yes) were assessed using logistic regression. Models were adjusted for age, sex, and highest household education. Differences in BMI z-scores between participants who were meeting versus not meeting the different combinations of recommendations were determined using the PROC DESCRIPT command and CONTRAST statement. Statistical significance was set at a *p* value of <0.05. The 95% confidence intervals (CIs) and coefficients of variation were derived using the bootstrap re-sampling method to account for the complex sampling design of the CHMS [26].

## Results

A total of 803 participants had complete data and were included in the present analyses (355 3-year-olds and 448 4-year-olds). Descriptive characteristics of the participants are presented in Table 1. In the full sample, 69.0% were in the healthy weight category. Participants were physically active for 4.6 h/day on average, of which approximately 1 h was spent in MVPA. Average screen time and sleep duration were 1.9 h/day and 10.6 h/day, respectively. No significant differences were observed between boys and girls for any descriptive characteristic, including movement-related behaviours.

**Table 1:** Weighted participant characteristics of the 2009/11, 2012/13 and 2014/15 Canadian Health Measures Survey

	Full sample (*n* = 803)		Boys (*n* = 400)^a^		Girls (*n* = 403)^a^	
	Mean	95% CI	Mean	95% CI	Mean	95% CI
Age (years)	3.5	3.5–3.6	3.5	3.4–3.6	3.5	3.4–3.6
Highest household education (%)						
Secondary school or less	14.9	11.7–18.8	10.8^E^	6.8-16.6	18.8	13.6–25.5
Some post-secondary	35.2	29.1–41.7	37.1	29.7–45.2	33.3	25.9–41.7
Bachelor’s degree or higher	49.9	43.5–56.3	52.1	43.5–60.6	47.9	40.4–55.4
Body weight status^b^ (%)						
Healthy weight	69.0	63.2–74.1	67.1	60.5–73.1	70.7	62.2–77.9
At risk of overweight	23.1	19.0–27.8	23.3	17.9–29.6	23.0	17.6–29.4
Overweight/obesity	7.9^E^	5.0-12.3	9.6^E^	5.7-15.7	F	–
Body mass index z-score	0.59	0.44–0.74	0.70	0.53–0.88	0.49^E^	0.24-0.73
Movement behaviours^c^						
MVPA (min/day)	68.1	65.4–70.7	69.5	66.6–72.3	66.7	63.2–70.2
LPA (min/day)	209.7	203.9–215.5	207.8	201.1–214.5	211.5	203.9–219.1
Total physical activity (min/day)	278.8	271.0–286.5	278.4	270.2–286.5	279.2	268.8–289.6
Screen time (h/day)	1.9	1.8–2.1	2.0	1.8–2.2	1.9	1.7–2.1
Sleep duration (h/day)	10.6	10.5–10.8	10.7	10.5–10.9	10.6	10.4–10.8

*CI* confidence interval, *LPA* light-intensity physical activity, *MVPA* moderate-to-vigorous physical activity

Data are presented as mean for continuous variables and percentages for categorical variables

^a^Data were not significantly different between boys and girls (all *p* > 0.05)

^b^Defined according to the World Health Organization criteria [24]

^c^Physical activity was based on accelerometer data, and screen time and sleep duration were parent-reported

^E^Use with caution (coefficient of variation >16.6%)

^F^Too unreliable to be published (coefficient of variation >33.3%)

**Fig. 1:**
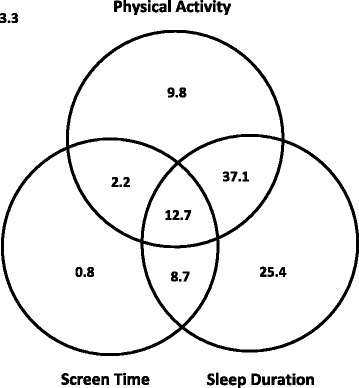
*Title:* Venn diagram showing the proportion (%) of participants meeting no recommendations; the physical activity, screen time, and sleep duration recommendations; and combinations of these recommendations, in the full study sample (*n* = 803) *Legend:* The recommendations are: ≥180 min/day of physical activity (of which ≥60 min is moderate-to-vigorous physical activity), ≤1 h/day of screen time, and 10–13 h/day of sleep. The sum of each circle is equivalent to the % meeting each individual recommendation (i.e., 61.8% for physical activity, 83.9% for sleep duration, and 24.4% for screen time).

Figure 1 illustrates the proportion of preschool children in the full sample who met the physical activity, screen time, and sleep duration recommendations, and combinations of these recommendations. A total of 12.7% met all three recommendations. The majority met the sleep duration (83.9%) and physical activity (61.8%) recommendations, while only 24.4% met the screen time recommendation. Overall, 3.3% of participants met none of the three recommendations.

Table 2 shows the odds ratios for “at risk of overweight, or overweight/obesity” associated with meeting versus not meeting the physical activity, screen time, and sleep duration recommendations (and combinations of these recommendations) in the full sample. Overall, none of the odds ratios were statistically significant. Likewise, none of the differences in BMI z-scores were statistically significant between preschool children meeting versus not meeting the different combinations of recommendations in the full sample (Table 3).

**Table 2:** Odds ratios for “at risk of overweight, or overweight/obesity”^a^ associated with meeting vs. not meeting the physical activity, screen time, and sleep duration recommendations and combinations of these recommendations in the full study sample (*n* = 803)

	OR	95% CI	*p* value
Meeting the following recommendations:			
At least PA	0.78	0.44–1.38	0.38
At least ST	0.64	0.37–1.08	0.09
At least SLEEP	1.52	0.75–3.05	0.24
At least PA + ST	0.66	0.34–1.31	0.23
At least PA + SLEEP	0.96	0.58–1.60	0.87
At least ST + SLEEP	0.73	0.39–1.35	0.30
All three recommendations	0.76	0.37–1.57	0.45

*CI* confidence interval, *OR* odds ratio, *PA* physical activity, *SLEEP* sleep duration, *ST* screen time

Models adjusted for age, sex, and highest household education

Meeting the recommendations is defined as ≥180 min/day of physical activity (of which ≥60 min is moderate-to-vigorous physical activity), ≤1 h/day of screen time, and 10–13 h/day of sleep

^a^Defined according to the World Health Organization criteria [24]. It was not possible to examine the overweight/obesity category only, as the numbers in that group were too low (8% of the sample)

None of the odds ratios are statistically significant

Physical activity was based on accelerometer data, and screen time and sleep duration were parent-reported

**Table 3:** Differences in BMI z-score between participants meeting vs. not meeting the physical activity, screen time, and sleep duration recommendations and combinations of these recommendations in the full study sample (n = 803)

	Total sample (n = 803)		Boys (n = 400)		Girls (n = 403)	
	BMI z-score	95% CI	BMI z-score	95% CI	BMI z-score	95% CI
At least PA						
Meet	0.59^E^	0.39–0.79	0.79	0.56–1.03	F	–
Do not meet	0.59^E^	0.38–0.80	0.55^E^	0.30-0.79	0.62^E^	0.33-0.92
At least ST						
Meet	0.41^E^	0.22–0.61	F	–	0.40^E^	0.13–0.66
Do not meet	0.65	0.47–0.83	0.78	0.59–0.98	0.52^E^	0.19-0.84
At least SLEEP						
Meet	0.59	0.43–0.76	0.63	0.45–0.80	0.57^E^	0.28-0.85
Do not meet	0.57^E^	0.21–0.93	1.16^E^	0.52-1.81	F	–
At least PA + ST						
Meet	0.41^E^	0.23–0.58	0.52^E^	0.19-0.85	F	–
Do not meet	0.62	0.45–0.79	0.73	0.54–0.92	0.52^E^	0.23-0.80
At least PA + SLEEP						
Meet	0.60^E^	0.38–0.82	0.69^E^	0.45-0.93	F	–
Do not meet	0.58	0.39–0.77	0.72^E^	0.44-1.00	0.46^E^	0.23-0.69
At least ST + SLEEP						
Meet	0.45^E^	0.22–0.67	F	–	0.45^E^	0.16–0.73
Do not meet	0.63	0.46–0.80	0.76	0.58–0.95	0.50^E^	0.18-0.81
All three recommendations						
Meet	0.46^E^	0.27–0.65	0.59^E^	0.21-0.96	F	–
Do not meet	0.61	0.44–0.78	0.72	0.54–0.90	0.50^E^	0.22-0.79

*BMI* body mass index, *CI* confidence interval, *PA* physical activity, *SLEEP* sleep duration, *ST* screen time

Meeting the recommendations is defined as ≥180 min/day of physical activity (of which ≥60 min is moderate-to-vigorous physical activity), ≤1 h/day of screen time, and 10–13 h/day of sleep

No significant differences in BMI z-scores were observed between meeting vs. not meeting the different recommendations

Physical activity was based on accelerometer data, and screen time and sleep duration were parent-reported

^E^Use with caution (coefficient of variation >16.6%)

^F^Too unreliable to be published (coefficient of variation >33.3%)

## Discussion

The objectives of this study were to quantify the prevalence of preschool-aged children in Canada meeting the new *24-Hour Movement Guidelines*, and examine the extent to which adherence to the specific recommendations, and different intermediate combinations, was associated with adiposity indicators. A key finding was that almost all (~97%) preschool children met at least one of the recommendations, but that very few (~13%) met all three recommendations. A large majority met the sleep duration recommendation (83.9%), followed by the physical activity (61.8%) and screen time (24.4%) recommendations. None of the combinations of recommendations were associated with adiposity in this sample of preschool-aged children.

The present findings are in line with those of Cliff et al. [27], who reported that, in a sample of 246 preschool-aged children in Australia, 14.2% met the integrated *24-Hour Movement Guidelines*. They also reported that high proportions of the children met the physical activity (93.1%) and sleep duration (88.6%) recommendations, and that a low proportion met the screen time recommendation (15.0%). Although they did not have information on adiposity in their study, they reported that preschool children who met more of the recommendations, especially the screen time and sleep duration guidelines, had better social cognitive development than those meeting no or fewer guidelines [27]. Lee and colleagues [28] and Santos and colleagues [29] reported similar findings in Canadian and Australian toddlers, respectively, in that high proportions met the physical activity and sleep guidelines, and low proportions met the screen time and overall guidelines. Thus, strategies and programs to promote adherence to the screen time recommendation among young children are needed but may be challenging to achieve in the current technophilic society. The American Academy of Pediatrics [30] recently provided several areas in which pediatric providers can offer specific guidance to families in managing their young children’s screen time use, not only in terms of content or time limits, but also emphasizing the importance of parent-child shared media use and allowing the child to take part in other developmentally healthy activities.

The fact that we did not find an association between movement behaviours and adiposity in this sample of preschool children is not very surprising. Kuzik et al. [31] published a systematic review on the relationships between combinations of movement behaviours and health indicators in children of the early years (0–4 years). While none of the 10 studies included in their review reported on all three combinations of movement behaviours, studies that looked at the combination of two movement behaviours generally found mixed findings for the associations with adiposity in this population, mainly based on very low- to low-quality evidence.

The observation that no association was found between combinations of movement behaviours and adiposity in preschool children is in contrast to what is reported for school-aged children and adolescents (5–17 years). Specifically, combinations of lower sedentary behaviour and higher physical activity have been shown to be favourably associated with adiposity in 8/8 studies included in a recent systematic review [32]. A possible explanation for this difference between age groups is the accumulative nature of adiposity, i.e., the prevalence of overweight and obesity increases as children progress from the early years to adolescence, and it may be too early to detect excess adiposity in prechool-aged children with small inter-individual variability in the data [33]. Other possible explanations include (i) differences in measurement tools to assess movement behaviours; (ii) differences in the robustness of study designs (e.g., smaller sample sizes in preschool children); (iii) the likelihood that it is more difficult to find associations with adverse health indicators in a younger and healthier population of children; or (iv) there is truly no relationship in this age group. Adiposity at this age may be more strongly determined by factors such as genetic makeup, growth, or diet than movement behaviours.

Physical activity, sedentary time, and sleep duration are three co-dependent behaviours that fall on the 24-h movement/non-movement intensity continuum [1]. Compositional data analyses provide an appropriate method for analyzing the association between co-dependent movement behaviour data and health indicators [2]. Carson et al. [34] recently employed compositional analyses to examine the integrated nature of movement behaviours and their relationship with adiposity in Canadian preschool-aged children. They observed null relationships for sleep, sedentary time, and physical activity relative to one another; however, the overall composition of movement behaviours was favourably associated with BMI z-scores, suggesting that the distribution of time between the movement behaviours across the whole day matters. Future research that employs appropriate analytic strategies for dealing with a finite geometry such as a 24-h day will be instrumental in our understanding of the implications of movement behaviours on various health outcomes in different populations, and will provide important information for public health guidance.

Strengths of this study include the representative sample of preschool children, the use of objectively measured (accelerometer) data for physical activity, and the fact that this is the first Canadian study to report on the proportion of preschool-aged children meeting the new *24-Hour Movement Guidelines*. The main limitations include the cross-sectional design (which precludes causal inferences being made), the lack of information about diet and timing of adiposity rebound, and the self-reported nature of screen time and sleep duration (which can result in misclassification). Accelerometer wear time was lower in respondents not meeting the physical activity recommendation (11.3 h/day) compared to those meeting it (12.3 h/day); however, wear time adjustment did not affect the odds ratios for “at risk of overweight, or overweight/obesity”. Also, not all types of screen devices were listed in the screen time questionnaire (e.g., smartphones and tablets were not part of the response options), screen time duration was reported in a categorical nature and therefore was not precise, and only the duration of movement behaviours was assessed. Other aspects of these movement behaviours, such as sleep disturbances, the content of screen time (e.g., educational or not), or the quality of physical activity (e.g., active outdoor play vs. active video gaming) may also play an important role. Additionally, other health indicators such as social, emotional, cognitive, or motor development may be more sensitive to the movement behaviours than adiposity at this age; however, these health indicators were not available in the CHMS for this age group.

## Conclusions

Collectively, only ~13% of Canadian preschool-aged children met the new *24-Hour Movement Guidelines* (which integrate recommendations for physical activity, screen time, and sleep duration), particularly because only 24% of them met the screen time guideline of ≤1 h/day. Furthermore, we observed that none of the movement behaviours, or combinations of movement behaviours, were associated with adiposity in this sample. Future research should focus on finding solutions for promoting better adherence to the screen time recommendation in particular. Future studies also need to include a broader set of health indicators (not only adiposity) in order to offer a better understanding of the relationships between meeting the new *24-Hour Movement Guidelines* and overall health.

## Abbreviations

BMI: Body mass index
CHMS: Canadian Health Measures Survey
CI: Confidence interval
cpm: Counts per minute
LPA: Light-intensity physical activity
MVPA: Moderate-to-vigorous physical activity
